# The Rural Masses of China

**Published:** 1883-11

**Authors:** 


### THE RURAL MASSES IN CHINA.

A FAMILY, C. D., consisting of eight persons, owns an acre and a half of land. The land was bought by the grandfather of the present head and has never been subdivided since nor added to. He grows about seventy bushels of rice and thirty-five of wheat and some vegetables and cotton besides worth altogether in money about $50. He has two nephews who work outside and bring home something to help, and in that way they get along, but they are very poor. He pays government land tax to the extent of $1.50 a year. He and all his neighbors wear native blue cloth, spun and woven in the family by the women from cotton grown by themselves. He never wore foreign cotton. The coat he had on (a well-worn affair) had been made two years previously, and it would last two years more. It served him at night as a coverlet as well as a coat by day.

Another family owned four acres odd, only part of which was suitable for rice culture. Their income was about 80 bushels of wheat and 150 of rice, about a fourth of which they could usually sell. They paid something over $3 a year as Government land tax. They also grew more cotton than they could use, and sold every year about $10 worth. They were better off than some of their neighbors, but never saved any money. They had fifteen mouths to feed. The foregoing cases are given because they represent fairly the average condition to be found in rural China. The greater number of cultivators probably belong to the class of tenants. Some say the proportion of tenants to peasant proprietors is as seven to three ; others put it as three to two ; but, whether tenant or proprietor, the condition of the cultivator is much the same—that is, it rarely rises above what is just enough for the bare necessaries of life. My own observations have been mostly confined to this and the adjoining provinces, and I exclude the cultivators of tea, silk, and opium, who, growing a commodity more and more in demand and easily transportable, are in a far better position than the ordinary peasant ; but, speaking for the greater part of China, I believe that I am not overstating the case in saying that for the working agricultural masses it is a daily hand-to-hand struggle with want. In a succession of good years they are very comfortable, and they have enough to eat and to wear, and they have few other wants ; but population is ever increasing up to the food limit, and when a bad year or two comes they die off by hundreds or thousands.

---

A Western paper says : "Sam Weldom was shot last night in the rotunda by Harry Parsons." About the worst place a man can be shot, next to the heart, is in the rotunda. It invariably proves fatal.

